# A semi-supervised learning framework for quantitative structure–activity regression modelling

**DOI:** 10.1093/bioinformatics/btaa711

**Published:** 2020-08-10

**Authors:** Oliver Watson, Isidro Cortes-Ciriano, James A Watson

**Affiliations:** Evariste Technologies Ltd, Goring on Thames RG8 9AL, UK; Centre for Molecular Informatics, Department of Chemistry, University of Cambridge, Cambridge CB2 1EW, UK; Centre for Tropical Medicine and Global Health, Nuffield Department of Medicine, University of Oxford, Oxford OX1 2JD, UK; Mahidol-Oxford Tropical Medicine Research Unit, Faculty of Tropical Medicine, Mahidol University, Bangkok 10400, Thailand

## Abstract

**Motivation:**

Quantitative structure–activity relationship (QSAR) methods are increasingly used in assisting the process of preclinical, small molecule drug discovery. Regression models are trained on data consisting of a finite-dimensional representation of molecular structures and their corresponding target-specific activities. These supervised learning models can then be used to predict the activity of previously unmeasured novel compounds.

**Results:**

This work provides methods that solve three problems in QSAR modelling: (i) a method for comparing the information content between finite-dimensional representations of molecular structures (fingerprints) with respect to the target of interest, (ii) a method that quantifies how the accuracy of the model prediction degrades as a function of the distance between the testing and training data and (iii) a method to adjust for screening dependent selection bias inherent in many training datasets. For example, in the most extreme cases, only compounds which pass an activity-dependent screening threshold are reported. A semi-supervised learning framework combines (ii) and (iii) and can make predictions, which take into account the similarity of the testing compounds to those in the training data and adjust for the reporting selection bias. We illustrate the three methods using publicly available structure–activity data for a large set of compounds reported by GlaxoSmithKline (the Tres Cantos AntiMalarial Set, TCAMS) to inhibit asexual *in vitro Plasmodium falciparum* growth.

**Availabilityand implementation:**

https://github.com/owatson/PenalizedPrediction.

**Supplementary information:**

Supplementary data are available at *Bioinformatics* online.

## 1 Introduction

High-throughput experiments allow for the characterization of the target-specific activity of thousands to hundreds of thousands of small molecules (Martis and Radhakrishnan, 2011; Phatak *et al.*, 2009). The structure–activity data generated from these experiments can be used to fit supervised learning models with the aim of then finding molecular structures that maximize an outcome of interest, such as target activity, cytotoxicity or lipophilicity (Cherkasov *et al.*, 2014).

Three major methodological issues are apparent in this approach. First, it is necessary to represent small molecules using a finite-dimensional vector representation, such as extended-connectivity fingerprint (Rogers and Hahn, 2010), which loses much of the information in the true underlying molecular structure related to bioactivity. Many different fingerprint representations are available, and thus methods that quantify relative information loss between different fingerprint representations are necessary to make an optimal choice. For instance, two molecules with uncorrelated bioactivity profiles might be close in fingerprint space depending on the fingerprint chosen (Muchmore *et al.*, 2008). Second, the accuracy of the predictive model degrades as the distance between the training and testing compounds increases (Netzeva *et al.*, 2005; Sheridan, 2015; Wallach and Heifets, 2018). The set of testing compounds for which the predictive value of the model is high is known as the applicability domain of the model (Netzeva *et al.*, 2005). This problem is sometimes taken into account by completely restricting models to the domain of compounds similar to those in the training set (Netzeva *et al.*, 2005). For general predictive purposes, however, it is desirable for the predictive model to properly account for this distance-dependent effect. Third, many structure–activity datasets have an inherent bias in that only molecules with a certain minimal target-specific activity are characterized and reported, e.g. Gamo *et al.* (2010). A bias towards active molecules will result in overly optimistic predictions of the activity values of new molecules, whereas models trained on datasets mostly comprising inactive molecules might hamper the discovery of structurally novel active compounds (Cortes-Ciriano *et al.*, 2018; Norinder and Boyer, 2017; Sun *et al.*, 2017).

In this work, we consider each of these three issues and provide methodological solutions. We use the Tanimoto distance as a metric on molecular space, which has proved suitable in quantifying molecular similarity in multiple drug discovery applications (Bajusz *et al.*, 2015). We show how Tanimoto distance can be used for a systematic comparison between different fingerprint representations. We show that it is possible to explicitly adjust model predictions for both the activity-dependent selection bias, and for the distance-dependent predictive degradation by accounting for the underlying geometry of molecular space. The adjusted predictions are made using a semi-supervised learning framework. This takes as input a set of labelled compounds (structures with labelled activity values) and a larger set of unlabelled compounds (only structures) which provide an empirical representation of the overall distribution of ‘feasible’ small molecules, that is amenable to synthesis and displaying drug-like properties (Matter *et al.*, 2012; Walters and Murcko, 2002). Semi-supervised learning refers to the set of methods developed in machine learning that use labelled (in this case structures with corresponding activity values) and unlabelled data (no corresponding activity values) to build predictive models, see for example, the studies by Käll *et al.* (2007) and Shi and Zhang (2011). The unlabelled data allow for a more accurate representation of the set of ‘feasible’ compounds that could have been part of the (unknown) screening process.

We illustrate this methodology on the Tres Cantos AntiMalarial Set (TCAMS), an open access screening dataset generated by GlaxoSmithKline based on the *Plasmodium falciparum* 3D7 asexual assay (Gamo *et al.*, 2010). We fit random forest and ridge regression models to these data. We use held-out data to compare the performance of the semi-supervised framework—which uses the unlabelled data and explicitly adjusts for Tanimoto distance between testing and training data—against the standard fully supervised framework.

## 2 Materials and methods

### 2.1 Definitions and notation

We use the following notation throughout. Compounds (small molecules) are denoted x∈X, where X is the unknown space of all feasible compounds. A compound *x* is represented by its ‘fingerprint’, a binary vector of some fixed dimension *p*. This vector representation of *x* is constructed via a ‘fingerprint mapping’, as described by Rogers and Hahn (2010), which uses a mathematical hash function to map the space of compounds into binary vectors of some finite dimension. Fingerprint mappings are not injective: two different compounds can have the same fingerprint (Cortes-Ciriano *et al.*, 2018; Rogers and Hahn, 2010). Identifying compounds by their fingerprint representation (for a given fingerprint mapping) allows us to define a metric over molecular space. We use the Tanimoto distance (also known as the Jaccard distance), defined as one minus the Tanimoto similarity (Bajusz *et al.*, 2015). The Tanimoto similarity of two compounds *x_i_* and *x_j_* is the number of substructures common to both compounds, divided by the total number of substructures that appear in at least one of the compounds (Bajusz *et al.*, 2015) [This is an approximation, since the hash mapping can map different substructures to the same bit index, see Rogers and Hahn (2010). Thus the presence of a ‘1’ in the same dimension for the fingerprints of two compounds means that they are likely to share some common substructure, but they do not conclusively do so.].

Written as Boolean operators on binary vectors, this is $\left|x_{i}\cap x_{j}|/|x_{i}\cup x_{j}\right|$. The rationale for choosing this metric is that only sharing a particular substructure provides information regarding similarity, and two compounds that share no substructures are thought of as being maximally different (for want of a better model for representing molecules in a finite-dimensional space). We denote the Tanimoto distance between compounds *x_i_*, *x_j_* as $d(x_{i},x_{j})$. For notational simplicity, we do not include the dependency on the underlying fingerprint mapping, though this mapping will in fact affect the distance *d*. In addition we define the setwise Tanimoto distance between a compound *x* and a set of compounds S as $d(x,S)={\text{min}}_{s\in S}d(x,s)$. This is the Tanimoto distance between *x* and its nearest neighbour in S. We note that for a finite dimension *p*, the set of feasible pairwise distances is discrete.

Our semi-supervised structure–activity regression modelling framework applies to the following set-up, whereby there are two distinct sources of data. First, we have labelled structure–activity data denoted $L_{n}={\left\{x_{i},y_{i}\right\}}_{i=1}^{n}$ (L for labelled), comprising *n* compounds, where *y_i_* is the response value for the compound *x_i_*. In our setting, *y_i_* is the target-specific activity of the compound *x_i_* for some pre-defined target of interest, but in general, it could represent other outcomes of interest (e.g. *in vitro* cytotoxicity, or lipophilicity). The response *y_i_* is a (unknown) function of *x_i_* and as such can be written $y_{i}=y(x_{i})$.

We examine the case where the labelled data has the following type of selection bias. The responses *y_i_* are all greater than a known cutoff value $L_{\min}$. We denote as ‘actives’ the molecules with a response value greater than $L_{\min}$, and as ‘inactives’ those less than $L_{\min}$. The unknown set of all active molecules is denoted A. The compounds *x_i_* in our structure–activity dataset $L_{n}$ are therefore a strict subset of A as they have been selected on the basis of observed activities $y_{i}>L_{\min}$. We assume that the set $L_{n}$ was derived by screening a larger set of compounds $L_{n\prime }$ (of known or unknown size n′>n), and then choosing the active compounds amongst them: $L_{n}=L_{n\prime }\cap A$. The critical point here is that the inactive compounds in the larger set $L_{n\prime }$ are unknown or unavailable for analysis.

Second, we have unlabelled structure data of size *N* denoted $U_{N}$ (U for unlabelled). By construction, there are no labelled compounds in $U_{N}$ ($U_{N}\cap L_{n}=\emptyset$). In general, in this set-up, it is assumed that n≪N, which is that of many semi-supervised learning problems whereby there is a smaller, well curated labelled dataset, and a much larger unlabelled dataset.

The key assumption that guides the following methodology is that the unlabelled data $U_{N}$ are sampled from the same data generating process as the unknown set of screened compounds $L_{n\prime }$. We note that this assumption is, in general untestable, however, we show how specific deviations can be detected and corrected for. It is worth noting that if we knew the inactive structures in $L_{n\prime }$ then much of the framework developed here would be unnecessary, but in practice the availability of large sets of active and inactive compounds for a target of interest is rather limited, thus possibly biasing predictive modelling applications in preclinical drug discovery.

### 2.2 Quantifying the utility of a fingerprint representation

The utility of a fingerprint mapping of small molecules in the context of modelling a specific response (outcome) can be quantified by characterizing the correlation between the responses *y_i_* (in our case activity) as a function of distance (using Tanimoto distance) for pairs of compounds. The following provides a non-parametric method for estimating the distance-dependent covariance of the activity of two compounds. This can be used as a general approach for visualizing the quality of a given *p*-dimensional fingerprint mapping.

In general, for any two compounds *x_i_*, *x_j_*, the joint distribution of their respective activities *y_i_*, *y_j_* can be estimated as $\left(\begin{array}{cc} \bar{y} & \sigma \\ \sigma & \bar{y} \end{array}\right)$, where $\bar{y}$ is the mean activity value, and *σ* is the covariance. If the distance metric over the fingerprint mapping of molecular space is a good representation of the true distance between molecules (and therefore, the true average difference in activities), then this covariance *σ* will be a function of the distance $d(x_{i},x_{j})$ and should be modelled accordingly.

With this aim, we define $B_{\delta }\subset L_{n}\times L_{n}$ as the set of all distinct pairs of active compounds for which the pairwise distance is exactly *δ*:

(1) $$B_{\delta }=\{x=(x_{i},x_{j}):\;d(x_{i},x_{j})=\delta ,x_{i}\neq x_{j}\}.$$

The set $B_{\delta }$ can then be used to empirically estimate the distance-dependent covariance function $\sigma^{2}(\delta )$:

(2) $$y(x_{i})-y(x_{j})∼N\left[0,\sigma {\left(\delta \right)}^{2}\right],\;x=(x_{i},x_{j})\in B_{d},$$

where N is the normal distribution.

In practice, we can partition the range of observed distances into *K* bins and compute $B_{\delta_{i}}$ for each bin *δ_i_*. A bootstrap estimate of the standard error around $\hat{\sigma }(\delta_{i})$ can be obtained by bootstrapping with replacement the individual compounds within $B_{\delta_{i}}$ (bootstrapping at the compound level, not the pairwise distance level).

### 2.3 Semi-supervised prediction model

#### 2.3.1 Prediction goal of semi-supervised framework

Using the two data sources $L_{n}$ and $U_{N}$, we wish estimate the probability that a new compound $x^{*}$ has an activity greater than some pre-specified threshold of interest *I* (where *I* is significantly greater than $L_{\min}$). For example, this threshold could represent an activity high enough to warrant further experiments. We note that in general a ranking based on tail probabilities (function of the mean and higher moments of the distribution) will differ from a ranking based on mean predicted values. We predict whether $y^{*}>I$ using a semi-supervised framework, whereby we condition on the distance between $x^{*}$ and the training data $L_{n}$. First, the modelling framework uses the labelled data $L_{n}$ to fit a supervised predictive model of *y* given *x*, using the fingerprint representation of $x\in L_{n}$ as a *p*-dimensional predictive variable. Second, the predictions made by the supervised model are adjusted using the additional information of the distance between $x^{*}$ and the training data. These adjustments also specifically account for selection bias in the training data, by conditioning on whether $x^{*}$ is an active molecule or not. It is important to note the following:

1. By construction, all the responses $y_{i}\in L_{n}$ have values greater than $L_{\min}$. Therefore, by regression to the mean, a general regression model will predict for any new compound a value greater than $L_{\min}$, regardless of the overall frequency of active compounds observed under the data generating process (approximated by n/n′).
2. Using our metric *d*, we can observe whether the active compounds $L_{n}$ are closer together than compounds drawn from the same data-generating process without selection bias. Assuming that $L_{n}$ was generated by taking the active compounds from a much larger set of compounds generated from the same process that generates the unlabelled data, we can use the inter-compound distances of $L_{n}$, compared to inter-compound distances of compounds from $U_{N}$ to estimate the rate at which the probability of being active varies as function of distance to the training data under the metric *d*.

Point 1 explains why it is necessary to adjust predictions with the background frequency of active molecules; point 2 implies that a metric on molecular space along with the unlabelled data $U_{N}$ provide key additional information as to whether a given molecule $x^{*}$ is active or not. Specifically, we can use the information on the distance between $x^{*}$ and the training data $L_{n}$ to inform the prediction of $y^{*}$.

The prediction goal is expressed as the estimation of:

(3) $$P\left[y^{*}\geq I|d(x^{*},L_{n})\right].$$

By the law of total probability, conditioning on whether $x^{*}$ is active (i.e. $y^{*}>L_{\min}$):

(4) $$\begin{array}{c} P\left[y^{*}\geq I|d(x^{*},L_{n})\right]=P\left[y^{*}\geq I|d(x^{*},L_{n}),x^{*}\in A\right]P\left[x^{*}\in A|d(x^{*},L_{n})\right] \end{array}.$$

The omitted second half of the sum

(5) $$P\left[y^{*}\geq I|d(x^{*},L_{n}),x^{*}\notin A\right]=0$$

is equal to 0 as, by definition, $y^{*}$ cannot be greater than *I* if $x^{*}$ is not in A.

In the next sections, we outline (i) the estimation of the distance dependent probability that $x^{*}$ is active: $P\left[x^{*}\in A|d(x^{*},L_{n})\right]$; and (ii) the estimation of the conditional probability that $y^{*}>I$: $P\left[y^{*}\geq I|d(x^{*},L_{n}),x^{*}\in A\right]$. We simplify the estimation of (ii) by breaking it down into the predicted expected value of $y^{*}$, and the predicted uncertainty around this expected value. Assuming a given parametric form for the predictive distribution of $y^{*}$, we can estimate $P\left[y^{*}\geq I|d(x^{*},L_{n}),x^{*}\in A\right]$. This can be done by fitting a predictive distribution (conditional on being active) using the active data we have—as explained in a section below—and then re-centring and re-scaling using the mean and variance estimates from the predictive distribution.

#### 2.3.2 Distance-dependent probability that $x^{*}$ is active

Applying Bayes rule:

(6) $$P\left[x^{*}\in A|d(x^{*},L_{n})\right]=\frac{P\left[x^{*}\in A,d(x^{*},L_{n})\right]}{P\left[d(x^{*},L_{n})\right]}$$

 

(7) $$=\frac{P\left(x^{*}\in A\right)P\left[d(x^{*},L_{n})|x^{*}\in A\right]}{P\left[d(x^{*},L_{n})\right]}.$$

We estimate Equation (7) by estimating each of its three components.

First, we estimate $P\left[d(x^{*},L_{n})|x^{*}\in A\right]$ using a *v*-fold ‘cross-prediction’-type procedure. For example, taking *v *=* *2, we randomly partition $L_{n}$ into 2 equally sized subsets $L_{n/2}^{1},L_{n/2}^{2}$. This partition gives a total of *n* setwise distances for each element of $L_{n/2}^{1}$ to the set $L_{n/2}^{2}$, and vice versa. By repeating this procedure *k* times, we obtain *kn* setwise distances which form an empirical distribution of $P\left[d(x^{*},L_{n/2})|x^{*}\in A\right]$. For *v *=* *2, this procedure estimates $P\left[d(x^{*},L_{n/2})|x^{*}\in L_{n}\right]$. The choice of *v* corresponds to a bias-variance trade-off. Taking *v *=* n* (a leave-one-out procedure) results in *n* datasets that are likely to be highly similar to one another, resulting in an empirical distribution of $P\left[d(x^{*},L_{n-1})|x^{*}\in A\right]$ with high variance. Lower values of *v* (e.g. *v *=* *2) de-correlate the sets used to estimate these setwise distances and result in a lower variance but with increased bias due to the smaller sample sizes. The optimal choice of *v* can be determined from multiple runs with different values of *v*, which allows for an assessment of the bias introduced by the finite sample size.

Second, the denominator $P\left[d(x^{*},L_{n})\right]$ can be estimated using the empirical distribution of setwise distances $d(x,L_{n})$, where $x\in U_{N}$. A sensitivity analysis with respect to the size of the set $L_{n}$ can be done by random samples of size n/2 elements from $L_{n}$.

Third, the marginal (prior) $P(x^{*}\in A)$, which is the overall fraction of active compounds in X, can be estimated in two possible ways. If the number of compounds screened to generate the dataset $L_{n}$ is known, then *n* over the number of compounds screened approximates the overall fraction of actives in X. Otherwise, it is possible to use a limit argument. We assume that compounds very close to an active compound are themselves active: formally this means that $\underset{d(x^{*},L_{n})\to 0}{\lim}P\left[x^{*}\in A|d(x^{*},L_{n})\right]=1$. Therefore:

(8) $$P\left(x^{*}\in A\right)=\underset{d(x^{*},L_{n})\to 0}{\lim}\frac{P\left[d(x^{*},L_{n})\right]}{P\left[d(x^{*},L_{n})|x^{*}\in A\right]}.$$

This relies on the ability to accurately estimate both terms in the ratio in Equation (8). We discuss this in Section 2.5.1.

#### 2.3.3 Distance-dependent degradation of predictive accuracy

In this section, we show how to estimate the mean and variance of the predicted value of $y^{*}$ as a function of the distance between $x^{*}$ and $L_{n}$, conditional on $x^{*}\in A$. After fitting a model *M* to the labelled data $L_{n}$, instead of using the ‘naive’ predicted expected value $M(y^{*}|L_{n})$ (and modelled uncertainty around this estimate), we formally account for degradation in predictive accuracy as a function of the distance $d(x^{*},L_{n})$. By estimating this distance-dependent decrease in model accuracy, we can correctly penalize model predictions to obtain a calibrated estimate of $P\left[y^{*}\geq I|d(x^{*},L_{n}),x^{*}\in A\right]$.

For a given distance δ∈[0,1], we assess the ability of our predictive model *M* to extrapolate at a distance *δ* from the training data by doing the following:

- We standardize the response values *y_i_* so that the model *M* is fit to approximately standard normal data.
- For each compound $x_{i}\in L_{n}$, we construct a subset of the labelled data, defined as all compounds at least *δ* units of distance from *x_i_*. This is denoted ${\bar{L}}_{i,\delta }=\left\{x\in L_{n}:d(x,x_{i})\geq \delta \right\}$. This is the complement of the *δ*-ball centred around *x_i_*.
- We fit the model *M* to the data ${\bar{L}}_{i,\delta }$ and compute the out-of-sample prediction ${\hat{y}}_{M_{i,\delta }}=M(x_{i}|{\bar{L}}_{i,\delta })$.

Here, M(a|B) denotes the prediction on compound *a* of the model *M* fit to data *B*. The *δ*-distance prediction ‘quality’ of the model *M* can be assessed by the set of residuals ${\left\{y_{i}-{\hat{y}}_{M_{i,\delta }}\right\}}_{i=1}^{n}$. The decrease in predictive ability as a function of the setwise distance to the training data can be quantified by estimating smooth functionals $\hat{\beta }(\delta ),\hat{\epsilon }(\delta )$, whereby:

(9) $$y_{i}∼N\left(\hat{\beta }(\delta ){\hat{y}}_{M_{i,\delta }},\hat{\epsilon }{\left(\delta \right)}^{2}\right).$$

The estimated standard deviation $\hat{\epsilon }(\delta )$ can be interpreted as 1 minus the distance-*d* R-squared of the model *M*. The conditional predictive distribution of the response $y^{*}$ can then be estimated as:

(10) $$y^{*}∼N\left(\hat{\beta }[d(x^{*},L_{n})]M(x^{*}|L_{n}),\hat{\epsilon }[d(x^{*},L_{n})]\right).$$

### 2.4 Data

To illustrate our predictive framework, we used the Tres Cantos Antimalarial Set (TCAMS) (Gamo *et al.*, 2010) as the labelled data $L_{n}$. These data comprise 13 533 compounds, selected on the basis that they inhibited the growth of *P. falciparum* 3D7 by at least 80% at 2 *μ*M concentration (in this context, this is the assay defining ‘active’ compounds and the threshold $L_{\min}$). Subsequently, in this article, we will follow standard drug-discovery convention and refer to the activity level of the active compounds in pIC50 units. In the context of this assay, the active compounds have pIC50 values greater than 5.7. This set of compounds was discovered by screening a library of 1 985 056 compounds (an active discovery rate equal to 0.68%) (Gamo *et al.*, 2010). The structures for the inactive compounds were not reported, and hence, the available structures correspond to only active compounds.

We constructed (see Section 2.5.1) unlabelled datasets $U_{N}$ with publicly available data from the Molport database after having removed all compounds with recorded activities in TCAMS (there were 2044 compounds in Molport with canonical fingerprints equal to compounds in TCAMS, which we count as identical in this case). This gave a total of *N *=* *7 228 997 compounds with no activity values (unlabelled). We treat this dataset as representative of ‘accessible chemical space’—and thus sampling a compound randomly chosen from this set as a ‘random compound’.

The key assumption used in the estimation of Equation (7) is that the set $U_{N}$ is sampled from the same data-generating process as the unknown set $L_{n\prime }$. This allows us to use $U_{N}$ to adjust for the inherent selection bias when training a supervised regression model on $L_{n}$.

The set of unlabelled data $U_{N}$ was provided with a certain ordering (a set of numbered files, each with approximately 500 000 compounds). This ordering was strongly correlated with the setwise distance to the 13 533 compounds in the TCAMS dataset (labelled data). The MolPort company could not provide a reason for this particular ordering of their data. It would seem likely that the database was compiled over time, and thus the earlier compounds in the list are those that are simpler to synthesize and thus more likely to appear in other high compound collections.

We standardized all chemical structures in all datasets described above to a common representation scheme using the python module standardizer (https://github.com/flatkinson/standardiser). Inorganic molecules were removed, and the largest fragment was kept to filter out counterions (Fourches *et al.*, 2010). To represent molecules for subsequent model generation, we computed circular Morgan fingerprints (Rogers and Hahn, 2010) for all compounds using RDkit (release version 2013.03.02) (Landrum, 2017). Specifically, we computed hashed Morgan fingerprints in binary format using the RDkit function *GetMorganFingerprintAsBitVect*, to return values in ${\left\{0,1\right\}}^{128}$.

We decided to use Morgan fingerprints as compound descriptors given the higher retrieval rates obtained with this descriptor type in comparative virtual screening studies (Koutsoukas *et al.*, 2014). The radius was set to 2, and we used two fingerprint lengths of 128 and 1024.

### 2.5 Statistical methods

#### 2.5.1 Distance-dependent probability of being active

The estimation of $P[x^{*}\in A|d(x^{*},L_{n})]$ is critical for the performance of the predictive model, see Equation (4). This probability is proportional to the functional:

(11) $$f_{n,N}(\delta )=\frac{P\left[d(x,L_{n})=\delta |x\in A\right]}{P\left[d(x,L_{n})=\delta \right]},$$

where δ∈[0,1]. An estimate ${\hat{f}}_{n,N}(\delta )$ of this functional should satisfy two properties:

1. For *δ* = 0:
  $${\hat{f}}_{n,N}(0)=\frac{1-\epsilon }{P(x^{*}\in A)}$$
  , where ϵ≪1 and depends on the granularity of the metric over molecular space.
2.
${\hat{f}}_{n,N}(\delta )$ is monotonically decreasing in δ∈[0,1].

To estimate ${\hat{f}}_{n,N}(\delta )$: (i) we generate random samples from the distribution $P\left[d(x,L_{n})=\delta |x\in A\right]$ (the numerator); (ii) we generate random samples from the distribution $P\left[d(x,L_{n})=\delta \right]$ (the denominator); (iii) we use these two sets of random samples to determine a smooth estimate of the ratio as a function of *δ*, such that the two properties specified above are satisfied. In this procedure, *γ* is the bandwidth parameter of the Gaussian kernel density used to estimate both probability densities for every value of *δ* (from the library *sklearn*, the function *KernelDensity* with default parameters).

The optimal value of *γ* is chosen as follows. First, we use the *v*-fold cross-prediction method to sample from $P\left[d(x,L_{n})=\delta |x\in A\right]$ with *v *=* *2 and *k *=* *5, giving a total of 66 635 samples (input to the numerator estimation). Second, we choose ten equally spaced distances *δ* in the range [0..0.45]. For each of these distances *δ*, we choose 10 samples of 100 000 points from the MolPort database using a specific sampling strategy explained below. We then use binary search to find the optimal bandwidth *γ* such that the estimated ${\hat{f}}_{n}$ satisfies the property ${\hat{f}}_{n}(0)=1.$. A sensitivity analysis to the choice *v *=* *2 (see Supplementary Fig. S1 in Supplementary Materials) showed that the bias introduced by estimating $P\left[d(x*,L_{n})|x*\in A\right]$ using $P[d(x*,L_{n/2})|x*\in A]$ does not affect the kernel density estimation (Supplementary Fig. S2 in Supplementary Materials).

This results in one hundred values for *γ*, and we take the median estimate $\hat{\gamma }$. We then use this $\hat{\gamma }$ to choose a value of *δ* such that samples chosen using this probability weighting, when smoothed with bandwidth *γ*, have $f_{n}(0)=1$. This gives us values (rounded) of *γ* (bandwidth) = 0.09 and *δ* (for use in our sampling strategy) = 0.15.

The structure of the Molport data $U_{N}$, whereby compounds early on in the numbering are much more likely to be close to the TCAMS dataset than those further on in the numbering motivates the following important sampling-type approach to choosing an appropriate subset of the data to use in fitting our estimate of $P\left[d(x,L_{n})=\delta \right]$. We generate sets of unlabelled data from $U_{N}$, whereby the sampling probability decays as a function of the index of the unlabelled data using the following crude approach. The Molport data are divided into 15 files, in increasing order (with 500 000 compounds per file, apart from the last which only has half this amount). For a given distance value *δ*, our sampling strategy goes as follows. We calculate the number of compounds with minimum distance *δ* to the TCAMS dataset, giving us $n_{\delta ,i}$ for i∈[0..14]. We sample from file *i* (without replacement) with probability $n(\delta ,i)/\sum_{j}\left(n_{\delta ,j}\right)$.

We used the python library *scikit-learn* (Pedregosa *et al.*, 2011) version 0.19.1 and functions with default parameter settings except where stated otherwise.

#### 2.5.2 Degradation of predictive accuracy

To calculate the distance-dependent degradation functions $\hat{\beta }(\delta ),\hat{\epsilon }(\delta )$ (Equation 9), we choose a uniform grid of 10 values of *δ* spanning the interval [0,1]. For each *δ* value on this grid, we calculated $\hat{\beta }(\delta ),\hat{\epsilon }(\delta )$ as per Equation (9) where the underlying regression models were random forests (RF) and ridge regression, respectively. We then used these ten estimates to interpolate smooth functions $\hat{\beta }(\delta )$ and $\hat{\epsilon }(\delta )$ by minimizing least squares deviation. The function is of the form $g(\delta )=a/(1+e^{-b\delta^{c}})$. This function *g* is continuous, strictly decreasing and non-negative over the interval [0,1], with three free parameters (*a*, *b*, *c*).

#### 2.5.3 Testing of predictive models

To benchmark the performance of the proposed predictive framework with respect to simpler alternatives, we designed testing experiments. Training and testing data were selected on the basis of quantiles of the distribution of the activity values (Watson *et al.*, 2019). In this set-up, all labelled data with activity values below a chosen activity quantile $q_{\text{train}}$ are used as training data, and all labelled data with activity values above a chosen activity quantile $q_{\text{test}}$ are used as part of the testing data. In particular, $q_{\text{train}}\leq q_{\text{test}}$. The complete testing set is then composed of these labelled data in addition to a set of 500 000 compounds randomly chosen from the unlabelled dataset (MolPort).

The thresholds used were $q_{\text{train}}=\{7.0,7.5\}$, and $q_{\text{test}}=\{7.5,8.0\}$. In the TCAMS dataset, there are 237 compounds with activity ≥7.5, and 170 compounds with activity ≥8.0. We denote $X_{q_{\text{train}}}$ as the training data defined by the cut-off $q_{\text{train}}$. We denote $\hat{M}(\cdot |X_{q_{\text{train}}})$ as the predictive model (in our analyses, random forests or ridge regression) fit to the training data $X_{q_{\text{train}}}$.

Each compound $x^{*}$ in the testing data is ranked according to the following four scores:

1.
$S_{0}(x^{*})=\hat{M}(x^{*}|X_{q_{\text{train}}})$ . This is the predicted mean value of $y^{*}$. This is the unadjusted base model.
2.
$S_{1}(x^{*})=\hat{\beta }\left[d(x^{*},X_{q_{\text{train}}}\right]S_{0}(x^{*})$ . This is the predicted mean value of $y^{*}$ scaled by the distance-dependent penalty factor $\hat{\beta }(\delta )$, where *δ* is the setwise distance of $x^{*}$ from the training data.
3.
$S_{2}(x^{*})=P\left[x^{*}\in A|d(x^{*},X_{q_{\text{train}}})\right]S_{1}(x^{*})$ . This score uses the additional reduction factor which is the probability that $x^{*}$ is active given its distance from the training data.
4.
$S_{3}(x^{*})=F\left[S_{2}(x^{*}),\sigma^{2}\left(d(x^{*},X_{q_{\text{train}}})\right)\right]P\left[x^{*}\in A|d\left(x^{*},X_{q_{\text{train}}}\right)\right]$ , where F(μ,σ,λ) is the predicted cumulative distribution function of $y^{*}$ with mean *μ* and variance $\sigma^{2}$. This is the full model as specified in Equation (4).

**Fig. 1.**
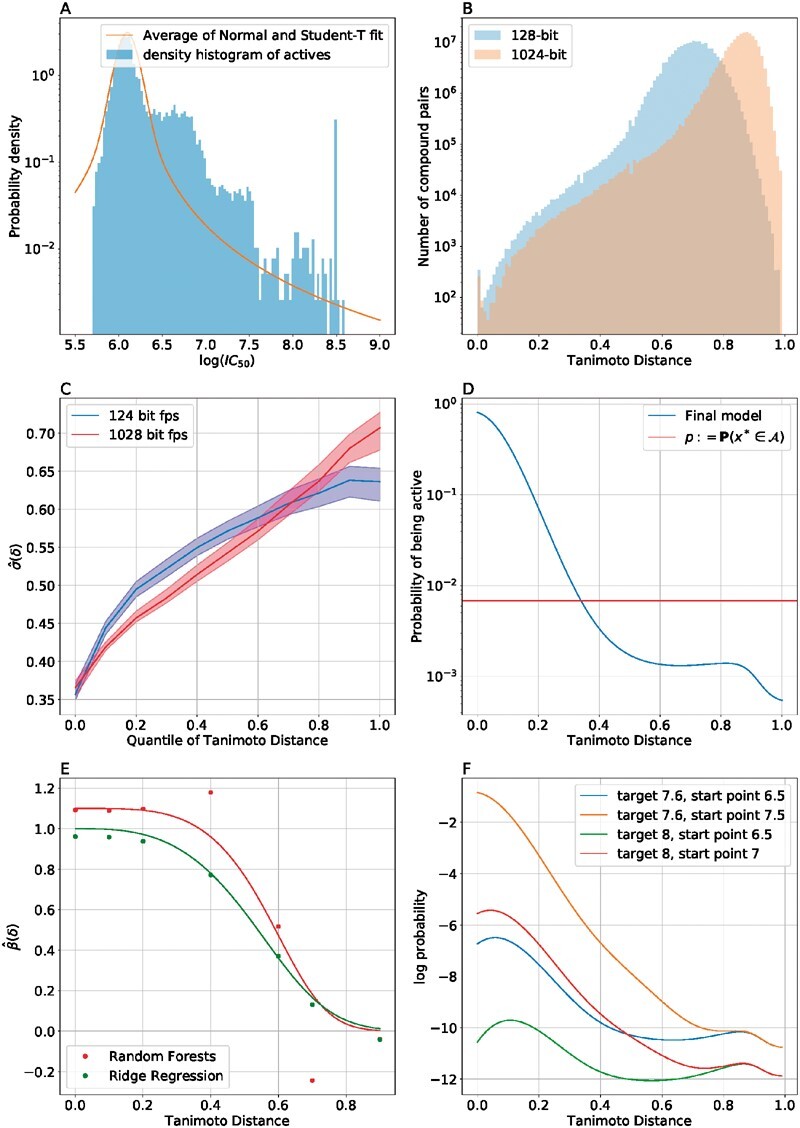
Visual representation of the activity data in TCAMS and overview of the model ingredients used for score *S*_3_. (**A**) Histogram of the distribution of the negative log (base 10) IC_50_ of the compounds in the TCAMs data (*n* = 13 533) with the *y*-axis on a logarithmic scale, with the estimated mixture distribution used in the prediction procedure overlaid (average of a normal and a student-*t* distribution); (**B**) histogram of the distribution of pairwise Tanimoto distances between molecules in the TCAMs dataset under a 128-bit fingerprint representation (blue) and a 1024-bit representation (orange); (**C**) non-parametric estimation of the Tanimoto distance-dependent activity covariance for both fingerprint representations (Equation 10). (**D**) The estimate of the fraction of compounds, which are active as a function of the minimum distance to a known active (baseline probability shown in red). (**E**) $\hat{\beta }(\delta )$ for Random Forests (Equation 9) and Ridge Regression. (**F**) Plot of the contour lines of the log probability of finding a target compound of activity *Z* at distance *d* from a starting compound of activity *W*

Figure 1Ashows the observed distribution of activities, which has a heavier tail than a Gaussian distribution. A Gaussian approximation of the observed activities gives a mean value of 6.25 and a standard deviation of 0.4, which implies that the expected number of compounds in the TCAMS dataset with activity ≥8 is 0.08, whereas in fact, there are 170 such compounds.

For the cumulative distribution function *F* in *S*_3_, we choose a mixture model which is a combination of a normal and a student-*t* distribution (shown in Fig. 1A as the orange line). We use the standard scikit-learn functions to fit a normal distribution to the activity data, and a student-*t* distribution to that same data. Our mixture model is then simply the average of these two distributions (This is an extremely crude way of fitting a normal and student-*t* mixture distribution, but as shown in the figure it suffices to capture the fact that activity distribution has a long right tail, while also capturing the bulk of the distribution.). We use this same distribution, but with the new values of *μ* and *σ* to do our calculations for *S*_3_. We implemented this fit using the inbuilt scipy fit functions, which fit distribution parameters to data. We took as our model the average of the Normal fit to the activity data and the student *t*-distribution fit to the data.

Finally, we choose our unlabelled data in one of two ways: ‘well-specified’ and ‘mis-specified’. This corresponding to choosing a set of unlabelled compounds using the sampling method described above, which are closer or further to the TCAMS data, respectively. In each case, we choose 500,000 unlabelled compounds. We use the same methodology as that used in calculating the fraction of actives to select the ’near’ dataset (recall, this consists in choosing from each file according to the number of compounds with minimum distance 0.19 from the TCAMS dataset). The ‘far’ dataset is chosen in the same way, but the fraction chosen from each file is the inverse of the number of compounds at that distance. This selection methodology aims to thus test the sensitivity of our results to the type of unlabelled data that the algorithm is searching over.

We then assess the performance of our selection methodologies $S_{0}..S_{3}$ in two ways. The first is an informal visual one. Given a selection methodology $S_{i},(i\in [0\ldots 3]$, we order the compounds in the complete testing set in decreasing order by their *S_i_* scores. We then plot the curve of the cumulative sum of the fraction of desired compounds found as we go through our ordered testing set. The ‘best’ method will give the curve that climbs highest fastest (since all curves start at 0 and end at 1), and we could formalize this if we wished by attaching a measure to each curve (e.g. the area under it). These plots summarize the more traditional metrics of ‘enrichment factors’ (EFs). Given a scoring system, the associated enrichment factor EF(p) for some threshold 0<p<1 is the number of desired compounds obtained by choosing the top scoring fraction *p* of the available data, divided by the number obtained when choosing the same fraction of the data at random. The maximal enrichment factor is just the maximum EF for any *p*. In our Supplementary Materials, we show the EFs for the standard thresholds (1% of data, 5% of data and the maximal EF).

#### 2.5.4 Limitations of methodology

A major limitation in the currently described methodology is that there is no propagation of uncertainty between the independent estimates. Further work would put this process into a fully Bayesian framework with uncertainty propagation. In addition, the solution to the estimation of the ratio $f_{n,N}(\delta )$ is only approximate and could possibly be improved.

## 3 Results

### 3.1 Methodological results

#### 3.1.1 Comparing combinations of fingerprints and metrics

A fingerprint mapping over compounds, together with a metric on the space of image of the map, induces a metric over molecular space. We provide a simple approach to compare the information content between different combinations of fingerprints and metrics. This can be used as a visual assessment of the quality of the induced metric on molecular space for the purpose of modelling a given target of interest. We note that the best combination of fingerprint and metric could be target dependent. The method consists of characterizing the distance-dependent covariance of the target between pairs of compounds. A metric over molecular space that does not preserve any information relating to the target activity would imply that the covariance between the activities of two compounds is independent of the distance between the two compounds. In reverse, if the expected covariance in target value between two compounds that are close together is much smaller than the expected covariance between random compounds we should see that the distance-dependent covariance is an increasing function (at least for small distance values) and the steeper the rate of increase, the more information the metric provides about values of the target. This also suggests a bootstrap approach to quantify the information content in a given metric over molecular space.

As illustration, we consider two related fingerprints, both extended-connectivity fingerprints of either 128 or 1024 features (bits). We use Tanimoto distance to construct an induced metric over molecular space. Figure 1C shows the distance-dependent covariance of the inhibition of asexual forms of *P.falciparum* in the TCAMs data. Because the units of distance for the two induced metrics on molecular space are different (as shown by Fig. 1B), we compare the covariance in terms of quantiles of distance, rather than absolute distance. This procedure generalizes to any fingerprint and metric pair, and tells us how the covariance of a given target changes as compounds move further away in molecular space in the metric under consideration.

This procedure can be used in two ways. First—simply to validate a choice of fingerprint, together with the Tanimoto metric, as a reasonable finite-dimensional representation for our purposes. Second, as a simple model to estimate the function $\hat{\sigma }(\delta )$. For a new compound $x^{*}$, the value $\hat{\sigma }(\delta_{x^{*}})$, where $\delta_{x^{*}}$ is the Tanimoto distance between $x^{*}$ and the nearest known compound, estimates the standard deviation around the predicted activity of $x^{*}$. We use this estimate in our score *S*_3_, as described in the Section 2.

#### 3.1.2 Distance-dependent degradation of predictions

For a given metric on molecular space that explains some of the variance of the target of interest (as discussed in the previous section), predictions of activity for a new compound $x^{*}$ should be informed by the distance between $x^{*}$ and the training data. The approach outlined in Section 2.3.3 provides a simple (although computationally expensive) procedure to incorporate this information into a model. The assumption is that for compounds sufficiently far away from the training data, the model should not provide any additional information for the target activities. Therefore, the output prediction should be the baseline value. The approach is to construct training sets that specifically test the ability of the supervised learning model to predict activity at a given distance *δ*. This allows us to test the assumption that predictive ability degrades as a function of the distance between compounds and to assess this degradation in predictive accuracy.

In the context of this article, we look at the degradation of predictive accuracy for models of the activity level (of active compounds) against *P.falciparum*. In Figure 1E, we show the estimates of the value $\hat{\beta }(\delta )$ that encodes this degradation for random forest and ridge regression models of the target value for a set of values of *δ*, together with our smoothed estimates derived from these.

The underlying intuition however (that regression models of any kind perform worse on compounds far away from the training set) is however quite general. In our Supplementary Results section, we show plots similar to Figure 1E in Supplementary Materials plot for 24 other protein targets (and ridge regression models). In all cases, we see approximately the same behaviour—the model predictions degrade as points become further away (Note that these plots do not correct for the degradation due to smaller numbers of data points in the fitted models.).

#### 3.1.3 Selection bias correction

We provide a method to correct for selection bias in training data. To correct for this bias in the training data, we need two extra sources of information:

- A set of unlabelled (no corresponding activity measurements) compounds which are assumed to have been sampled under the same data-generating process as the labelled compounds, but without activity-dependent reporting bias.
- An estimate of the background frequency of the discovery of active compounds under the data-generating process.

Given these two extra sources of information, we can use the Bayes rule to estimate the probability that a random compound is active, as a function of its minimum distance to a known active compound. In Figure 1D, we show our estimate of the probability of a random compound being active as a function of its distance to the nearest known active compound, together with the background rate for comparison.

The intuition behind the approach can be understood through the following analogy. Suppose we have a map where the observed activity value for a given point on the map is the altitude above sea level. Suppose we want to estimate how ‘jagged’ the terrain is, where jagged measures how quickly altitude changes between neighbouring points. Suppose further that many observations have been made uniformly at random across the map, but only those with an altitude greater than a given threshold were recorded. If the recorded points are clustered together, this implies that the terrain is divided into low and high regions; in other words, altitude varies smoothly. If on the other hand, the recorded points are not distinguishable from a set of points chosen uniformly at randomly on the map, this would indicate extremely jagged terrain. In our context, the Tanimoto distance puts all compounds onto a finite-dimensional space corresponding to this map. The unlabelled compounds are used to estimate the data-generating process, i.e. an estimate of how compounds are sampled across the ‘map’. This sampling procedure is very different from a uniform distribution. By comparing the pairwise distances between the active compounds (recorded points) to the pairwise distances between ‘random’ compounds (unlabelled data), we can estimate how smoothly the activity varies as a function of distance to the active compounds.

#### 3.1.4 Creating the selection score *S*3

Our framework does the following:

1. We determine the probability of being active as a function of the distance to the training data, as given by Equation (7).
2. We determine how the predictive accuracy of the model degrades as a function of the distance to the training data (Equation 9).
3. Determine how the covariance of the activity of two active elements varies as a function of the distance between them.
4. Given some model for the full distribution of activity values of the active compounds (as a function of variance and expected activity level)—put the above three steps together to compute, for any unknown compound, the full posterior distribution of its activity.

Figure 1F merges these components and illustrates how the fully adjusted model (score *S*_3_) works. We confine our attention to the random forest model. Rather than trying to plot the full predictive distribution as a function of *δ* for some compound (which would thus be a surface), we plot probability contour lines. Given some unknown compound *x*, at distance *δ* to the active training set, suppose that $S_{0}(x)(=\hat{M}(x^{*}|X_{q_{\text{train}}}))$ is the simple estimate from the random forest model (without any adjustment of any kind) for the activity of *x*. We call this value the ‘start point’. Given some target level of activity *T*, we wish to plot the log probability that y(x)>=T as a function of *δ*. We compute the probability that x∈A as a function of *δ*. Then, assuming x∈A, we compute the distribution of *y*(*x*). For this, we require three items:

1. The mean predicted value μ(δ):=E[y(x)|x∈A]. This is the score $S_{1}(x)$, which is $S_{0}(x)$ adjusted towards the mean activity level as a function of *δ*.
2. The variance of the predicted value $\sigma^{2}(\delta ):=E\left[{\left(y(x)-y(a)\right)}^{2}\right]$ where a∈A is the closest compound to *x*. We obtain this from the potency covariance plot (bottom left in Fig. 1) as a function of *δ*.
3. The distribution of y(x)|x∈A as a function of the mean μ(δ) and the variance $\sigma^{2}(\delta )$. Here, we use the mixture distribution as shown in panel A of Figure 1, but with our new estimates of μ(δ) and σ(δ). Once we have the distribution, we can compute the probability mass that lies above *T*.

### 3.2 Application to *P. falciparum* screening data

We analysed structure activity data on 13 533 compounds that were selected on the basis of inhibiting *P. falciparum* 3D7 asexual growth by more than 80% at 2 *μ*M (Gamo *et al.*, 2010). To assess the benefit of the semi-supervised framework, we compared the predictive performance between the derived semi-supervised predictive model (score *S*_3_) and the standard fully supervised predictive model that does not use the unlabelled data (score *S*_0_). Scores *S*_1_ and *S*_2_ are intermediate versions of the semi-supervised framework. The comparison between predictive frameworks (i.e. scores) was done using quantile-activity splitting (Watson *et al.*, 2019). This uses all compounds with activity below a certain threshold as training data, and all compounds with activity above a certain threshold as testing data.

We fit random forests and ridge regression models to two separate training sets: all compounds with activity less than 7 pIC50 and all compounds with activity less than 7.5 pIC50. Two separate testing sets were used: all compounds with activity greater than 7.5 pIC50 (*n *=* *237), and all compounds with activity greater than 8 pIC50 (*n *=* *170). The predictive performance of each fitted model was then assessed under four different predictive frameworks (scores *S*_0_ to *S*_3_, see Section 2.5.3).

**Fig. 2.**
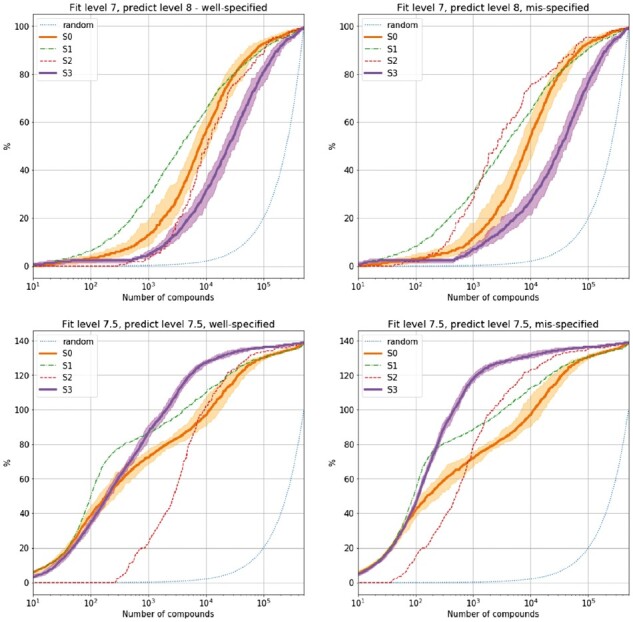
Comparison of predictive scores whereby random forests is the underlying predictive model. Here, the *y*-axis is the percentage of desired compounds found within the first *x* compounds ordered by the selection methodology. For methods *S*0 and *S*3, we include bootstrap 95% error thresholds from multiple data samples

**Fig. 3.**
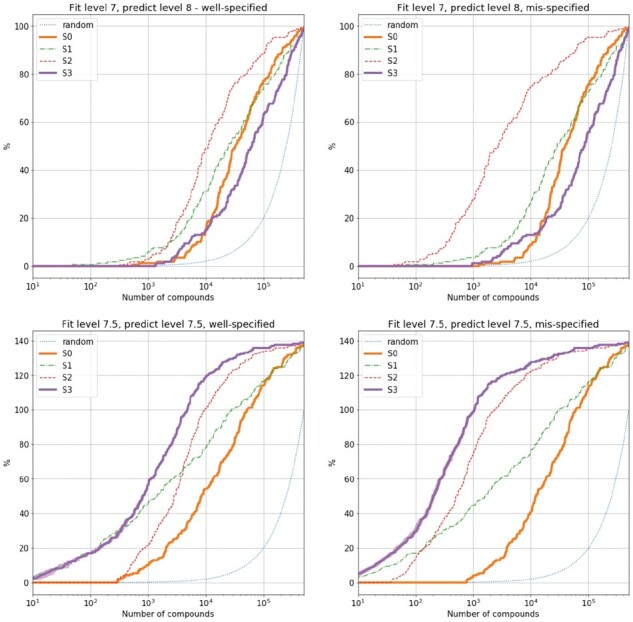
Comparison of predictive scores whereby ridge regression is the underlying predictive model. Here, the *y*-axis is the percentage of desired compounds found within the first *x* compounds ordered by the selection methodology. For methods *S*0 and *S*3, we include bootstrap 95% error thresholds from multiple data samples—though these are barely visible

A comparison of these four predictive frameworks is shown in Figure 2 for random forests and in Figure 3 for ridge regression. For simplicity, we show the results when training on compounds with activity (all in pIC50 units) less than 7 and testing on compounds greater than 8 (upper panels); and when training on compounds with activity less than 7.5 and testing on compounds with activity greater than 7.5 (lower panels). Each panel shows the percentage of true compounds (compounds in the TCAMS data not used in the model training stage and known to have activity above the desired threshold) discovered as a function of the number of compounds chosen from the testing set (500  000 compounds in total). In Supplementary Materials, we show the enrichment factor (the number of desired compounds found for a given selection methodology, divided by the number of desired compounds found if selecting at random) at various thresholds (1% of data, 5% of data and maximum enrichment factor achieved at any point). For a choice of 1000 compounds—a reasonable size for a drug discovery project—the naive model (score *S*_0_) performs consistently worse across all experiments that the full predictive framework (score *S*_3_). For example, in the most difficult testing scenario, where the training data are all compounds with activity less than 7, and the testing compounds are those with activity greater than 8, then *S*_3_ has much higher enrichment factors (particularly maximum enrichment factor, and particularly for ridge regression).

## 4 Discussion

The goal of this work was to provide methodological advances that lead to two improvements in the predictive ability of general quantitative structure–activity relationship regression models. First, for any given testing compound, the predicted activity should be ‘sensible’. By sensible we mean a distance-dependent regression to the mean response value. This implies that the model should predict the background expected response value for compounds whose structures are entirely different to the training compounds. This leads to having a model whose predictions are partly based on the background discovery rate of ‘active’ compounds (which is context specific) and the mean activity of these ‘active’ compounds. Second, the model predictions should be ‘useful’. By useful we mean that the adjusted model should outperform a ‘naive’ model at distinguishing ‘good’ compounds. We use a quantile-activity split approach to set up model testing experiments.

We investigate these two goals in the context of the TCAMS dataset. These two goals appear to be well aligned but they are not easy to jointly satisfy. For instance, the non-adjusted (‘naive’) random forest model (score *S*_0_), only using the labelled data, performs almost as well as the fully adjusted model (score *S*_3_) in identifying high-activity compounds in the testing data (Fig. 2). However, the non-adjusted model does not make sensible predictions overall, since it predicts a non-negligible asexual activity against *P.falciparum* 3D7 for any input compound (no distance-dependent regression to the mean). Method *S*_2_ does make sensible predictions by correctly predicting the average activity values for all compounds (due to the distance-dependent adjustment), but under-performs with respect to *S*_0_ substantially in three out of four testing experiments.

We show that these two goals can be achieved by explicitly modelling the full distribution of our prediction, rather than just the mean value, and taking this distribution into account in the optimization process. The method that does this (*S*_3_ in Figs 2 and 3) is the top performing method for choosing compounds overall. It is the top performing method in four of the eight tests performed, and no other method consistently dominates it (the closest is method *S*_1_, which, like *S*_0_, does not make sensible predictions overall).

The utility of having a general predictive model framework that satisfies both of these goals is that it opens up new questions for quantitative analysis, and in particular optimization. For optimization algorithms to converge, they need not only to produce accurate answers on the domain of interest (what we call a ‘useful’ model), but they also need to provide at least approximately correct answers outside that domain (what we call a ‘sensible’ model). In our testing experiments, all the methods tested (*S*_0_ to *S*_3_) provide rankings of all compounds. However, the fully adjusted model (score *S*_3_) has an additional advantage. The rank it provides for a given compound is derived from the probability that the compound will have an activity above a threshold of interest. Thus given three compounds $x_{0},x_{1},x_{2}$, with $S_{3}(x_{0})>S_{3}(x_{1})>S_{3}(x2)$, we can ask the question ‘would we have a higher chance of finding at least one compound with an activity above the threshold of interest if we tested *x*_1_ and *x*_2_, rather than just *x*_0_?’ This question cannot be answered by the other model adjustments, and this example can of course be extensively generalized. Most of the practical questions that face researchers in this area can be phrased in terms of trade-offs, e.g. ‘how many compounds should we make in one batch?’; ‘how similar should they be?’; ‘is it worth making one expensive compound that is predicted to be highly active, or testing ten cheap ones that are not predicted to be quite as good?’ (Huggins *et al.*, 2011; Valler and Green, 2000). We hope that this approach will make predictive models substantially more useful to practitioners.

## Supplementary Material

btaa711_Supplementary_Data
