# Supplementary material for: A semi-supervised learning framework for quantitative structure–activity regression modelling
Source: Bioinformatics. 2020 Aug 10;37(3):342–50. doi: 10.1093/bioinformatics/btaa711 (PMC8058768; doi:10.1093/bioinformatics/btaa711)
Supplement: btaa711_Supplementary_Data [file btaa711_supplementary_data.pdf]

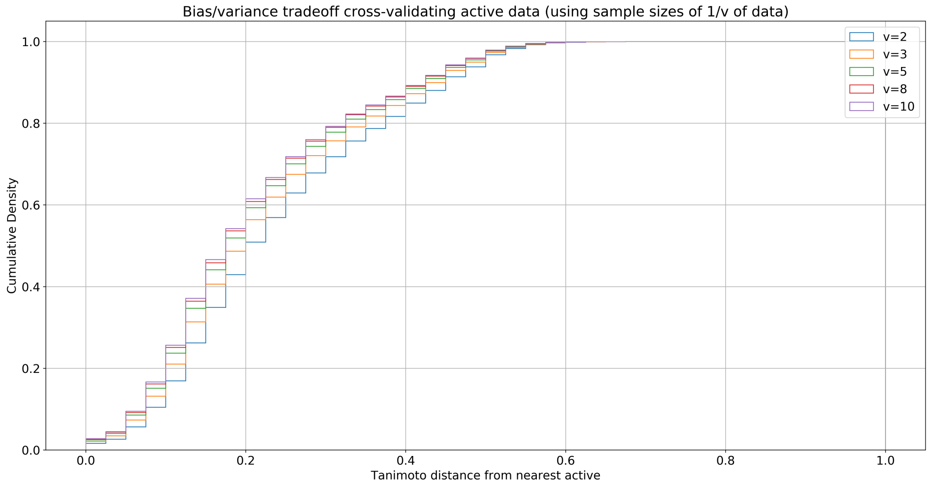

Figure 1: Plot of the cumulative density histogram of minimum distance of actives to known actives, using cross-validation for various v.

1 Supplementary results

1.1 Estimation of active probability

In Section 2.5.1 of the main paper, we assert that using a v-fold cross-validation with $v = 2$ introduces an acceptably small level of bias into our estimation of the likelihood of being active as a function of distance to the nearest know active. To support our claim we therefore show two plots. The first, Figure 1, shows the cumulative density histograms of the values $d(x - \mathcal{L}_n \setminus X), x \in X$, where $X \subset \mathcal{L}_n, |X| = n/v$. The second plot is the marginal version (corresponding to the pdf rather than the cdf) after having been smoothed with a Gaussian Kernel of bandwidth 0.05. This latter function (when scaled by $P(x \in A)$) is our estimate of the numerator of equation 7 in the main paper.

1.2 Enrichment Factor tables

In our table 1 we show the numerical enrichment factors corresponding to the plots in Figures 1 and 2 of the main paper. Note the following (non-standard) use of terminology. Enrichment factors are standardly given for a particular fraction of the data (usually 1% and 5%). We give these, but we also provide the maximal enrichment factor (EF max in our table), which is the largest enrichment factor found for any fraction of the data. This should not be confused with the maximal enrichment factor *for a given fraction of the data* - which refers to the enrichment factor which would be obtained at that fraction by a perfect scoring system.

1.3 Distance dependent predictive accuracy on alternative protein targets.

At the start of this paper we claimed that our methodology for estimating the distance dependent predictive accuracy, which we have only examined thus far in the case of our malaria activity, was quite general. We wish to provide some justification for this claim, and have therefore taken 24 of the compounds in [1], using 128-bit fingerprints and ridge regression only, to illustrate our point. Again we see that models are roughly well-calibrated at distance 0 from the training set, but become completely uninformative once we move sufficiently far away from it.

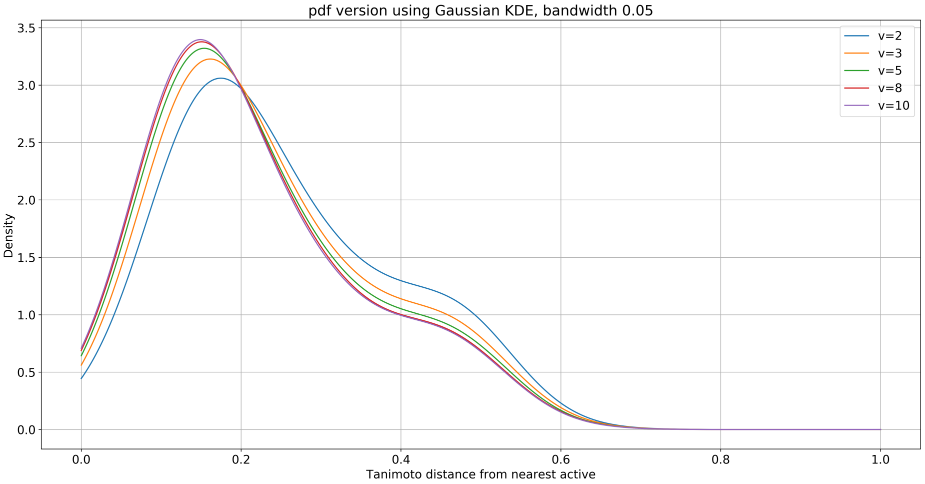

Figure 2: Density version of Figure 1, after having been smoothed with a Gaussian Kernel of bandwidth 0.05.

References

[1] Watson, Oliver P and Cortes-Ciriano, Isidro and Taylor, Aimee R and Watson, James A (2019) A decision-theoretic approach to the evaluation of machine learning algorithms in computational drug discovery *Bioinformatics*, **199**.

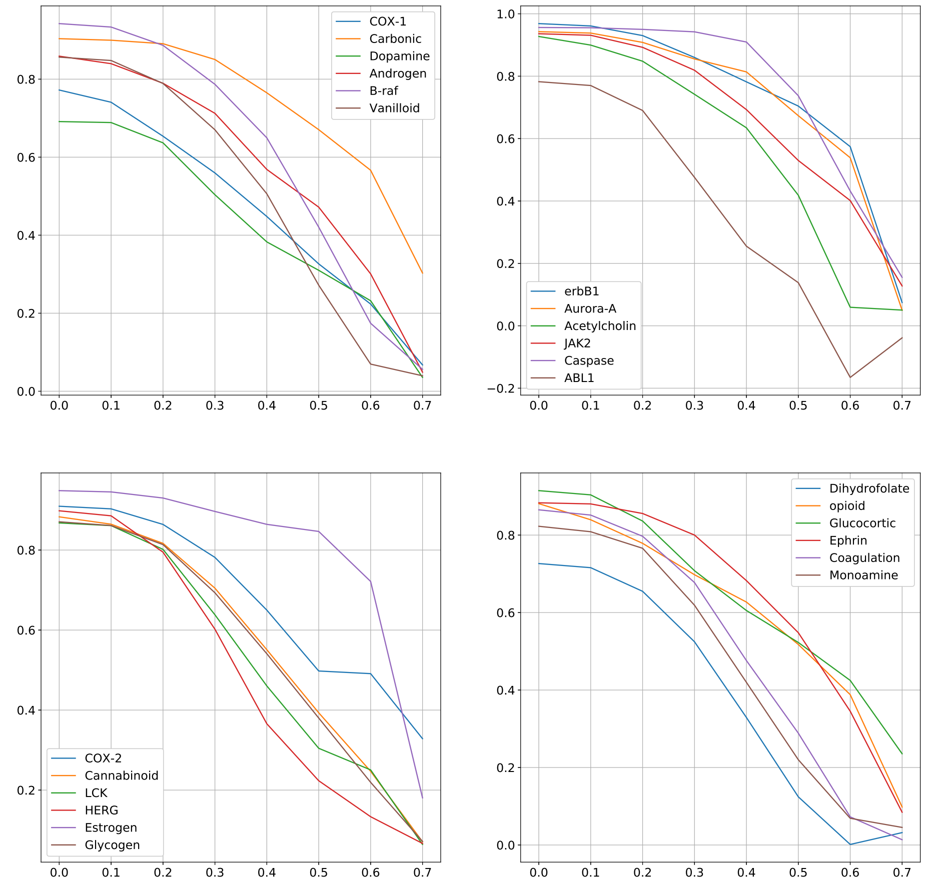

Figure 3: Demonstration of the distance-dependent degradation of predictive accuracy on 24 protein targets. The x-axis is Tanimoto distance from training set, and the y-axis is $\hat{\beta}(\delta)$ as defined in equation 9 of the main paper.

Table 1: Selected enrichment factor (EF) results corresponding to Figures 2 and 3 of the main paper. We used two prediction models, ridge regression and random forests (rf). The training data were all compounds with activity less than the training threshold; the testing data were all compounds with activity greater than the testing threshold and 500,000 unlabelled compounds (assumed to have low activity).

Prediction model	Score	Specification	Training threshold	Testing threshold	EF 1%	EF 5%	EF max
ridge	S0	well	7.50	7.50	25.42	10.85	40.34
ridge	S0	mis	7.50	7.50	18.22	10.08	19.82
ridge	S0	well	7.00	8.00	6.51	8.05	9.28
ridge	S0	mis	7.00	8.00	1.78	6.75	7.12
ridge	S1	well	7.50	7.50	49.15	14.15	1,445.19
ridge	S1	mis	7.50	7.50	47.87	13.47	1,004.03
ridge	S1	well	7.00	8.00	18.93	10.30	60.40
ridge	S1	mis	7.00	8.00	13.61	9.47	39.46
ridge	S2	well	7.50	7.50	55.08	17.29	89.77
ridge	S2	mis	7.50	7.50	80.92	18.56	591.96
ridge	S2	well	7.00	8.00	27.81	14.32	30.97
ridge	S2	mis	7.00	8.00	61.53	16.45	190.34
ridge	S3	well	7.50	7.50	74.14	18.90	820.50
ridge	S3	mis	7.50	7.50	89.39	19.15	1,607.98
ridge	S3	well	7.00	8.00	24.85	9.82	99.31
ridge	S3	mis	7.00	8.00	23.08	8.76	274.03
rf	S0	well	7.50	7.50	61.01	16.95	2,119.61
rf	S0	mis	7.50	7.50	63.13	17.12	2,119.61
rf	S0	well	7.00	8.00	33.13	14.79	227.66
rf	S0	mis	7.00	8.00	31.36	14.56	328.84
rf	S1	well	7.50	7.50	74.14	17.88	2,119.61
rf	S1	mis	7.50	7.50	76.68	17.97	2,119.61
rf	S1	well	7.00	8.00	46.74	14.79	643.38
rf	S1	mis	7.00	8.00	46.74	14.79	710.29
rf	S2	well	7.50	7.50	57.62	17.54	100.93
rf	S2	mis	7.50	7.50	81.34	18.90	652.19
rf	S2	well	7.00	8.00	27.81	14.56	30.89
rf	S2	mis	7.00	8.00	62.13	16.45	183.12
rf	S3	well	7.50	7.50	86.00	19.24	2,119.61
rf	S3	mis	7.50	7.50	93.63	19.58	2,119.61
rf	S3	well	7.00	8.00	38.46	13.96	532.72
rf	S3	mis	7.00	8.00	40.23	13.37	963.57
